# 

**Published:** 1904-09-15

**Authors:** 


					﻿THE
DENTAL REGISTER
EDITED BY
NELVILLE S. HOFF, D. D. S„
ANN ARBOR, MICH.
_ ___ ___ '
Vol. LVITI: ^PJFEMBER 15, 1904.	No. 9.
PRICE, ONE DOLLAR A YEAR IN ADVANCE.
PUBLISHED MONTHLY BY
SAMUEL A. CROCKER & CO.,
THE OHIO DENTAL AND SURGICAL DEPOT,
3» to 39 W. “tli SSL., Ciueiiiuriti, O.
Entered at the Postoffice at Cincinnati, 0., as second-class mail matter.
				

